# Comparative assessment of sarcopenia screening tools for patients with bone tumors: insights for enhanced clinical application

**DOI:** 10.3389/fnut.2025.1584706

**Published:** 2025-08-04

**Authors:** Jun Yu, Qiongyao Guan, Yanyu Chen, Lan Zhou, Jian Chen, Yingchun Zeng

**Affiliations:** ^1^Department of Bone Tumor, The Third Affiliated Hospital of Kunming Medical University, Yunnan Cancer Hospital, Kunming, China; ^2^School of Nursing, Kunming Medical University, Kunming, China; ^3^Department of Nursing, The Third Affiliated Hospital of Kunming Medical University, Yunnan Cancer Hospital, Kunming, China; ^4^Department of Nutrition, The Third Affiliated Hospital of Kunming Medical University, Yunnan Cancer Hospital, Kunming, China; ^5^Alice Lee Centre for Nursing Studies, Yong Loo Lin School of Medicine, National University of Singapore, Singapore, Singapore

**Keywords:** sarcopenia, screening tool, sensitivity and specificity, bone tumor, Chinese patients

## Abstract

**Background:**

Sarcopenia screening in bone tumor patients is challenging due to limited awareness, complex procedures, and high costs-especially since most research targets older adults, overlooking younger patients in this group. This study aims to compare the screening efficacy of five different tools for sarcopenia, and to identify the most appropriate screening tool for patients with bone tumors.

**Methods:**

The five sarcopenia screening tools assessed were SARC-F, SARC-Calf, SARC-F + EBM, and the Chinese versions of the Mini-Sarcopenia Risk Assessment scales (MSRA-5 and MSRA-7). The 2019 Asia Working Group for Sarcopenia (AWGS) criteria served as the reference standard for sarcopenia screening.

**Results:**

Among 300 bone tumor patients, 26% were found to have sarcopenia based on AWGS 2019 criteria. The screening tools varied in performance, with SARC-Calf showing the highest sensitivity and MSRA-7 the lowest specificity. Positive and negative predictive values were moderate across tools, with combined screening methods generally improving sensitivity. The highest overall accuracy (AUC) was observed when using a combination of SARC-F, SARC-Calf, and EBM, which provided both high sensitivity and acceptable specificity.

**Conclusion:**

The SARC-Calf and SARC-F + EBM tools demonstrated high accuracy in screening sarcopenia among bone tumor patients. The combined use of SARC-F, SARC-Calf, and SARC-F + EBM yielded superior screening performance, making them suitable for preliminary sarcopenia screening in this patient population.

## 1 Introduction

Bone tumors originate from bone tissue or other components within the bone, encompassing primary bone tumors as well as metastases from other malignancies, most commonly from the lungs and breasts. Bone metastases affect over 1.5 million cancer patients globally and spread through the blood or lymphatic system (1). Primary bone tumors, although rare, account for approximately 2–3% of all cancers and are frequently located at the ends of long bones, such as the distal femur, proximal tibia, and proximal humerus (2). In China, an estimated 24,200 bone tumor cases were reported in 2015, representing about 0.62% of all cancers (3). Factors such as chemotherapy, immunodeficiency, tumor-induced catabolism, fear of movement, pain, and poor sleep contribute to an increased risk of muscle loss in bone tumor patients (4, 5).

Sarcopenia is a progressive, systemic condition characterized by the loss of skeletal muscle mass, strength, and physical performance (6). The prevalence of sarcopenia varies according to diagnostic criteria and environmental factors, ranging from 5.5 to 25.7% (7). Among cancer patients, sarcopenia prevalence can be as high as 43% (8), significantly impacting clinical outcomes, such as increased chemotherapy toxicity, higher rates of complications, prolonged hospital stays, reduced quality of life, and elevated mortality risk (6, 9).

The diagnosis of sarcopenia is challenging due to variability in measurement techniques and diagnostic thresholds. Commonly used methods include computed tomography (CT), magnetic resonance imaging (MRI), dual-energy X-ray absorptiometry (DXA), ultrasound, and bioelectrical impedance analysis (BIA) (6). While CT and MRI are considered the gold standards for assessing muscle mass, their high costs, limited accessibility, and need for specialized personnel restrict their routine use (10). DXA provides inconsistent results across different instrument brands, and ultrasound lacks standardized cutoff values (11, 12). BIA, although affordable, is affected by factors such as body composition and fluid retention.

Given these limitations, screening tools like the SARC-F (strength, assistance with walking, rise from a chair, climb stairs and falls), SARC-CalF (SARC-F combined with calf circumference), EBM (elderly and body mass index information), Mini sarcopenia risk assessment-5 (MSRA-5), and Mini sarcopenia risk assessment-7 (MSRA-7) have gained attention as alternatives for early detection of sarcopenia (7). The 2018 European Working Group on Sarcopenia in Older People (EWGSOP) and the 2019 Asian Working Group for Sarcopenia (AWGS) recommend using screening tools to identify individuals at risk of sarcopenia to facilitate timely intervention (6, 13). However, these tools have primarily been validated in older adults and patients with other chronic conditions (1). Research on sarcopenia screening in cancer patients, especially those with bone tumors, remains limited, necessitating further investigation into the efficacy and applicability of these tools in this study population. Therefore, this study aims to compare the screening efficacy of five different tools for sarcopenia, and to identify the most appropriate screening tool for patients with bone tumors.

## 2 Materials and methods

This cross-sectional study was conducted at a provincial cancer hospital between August 2023 and November 2023. It is part of a broader investigation into the development and application of a risk prediction model for sarcopenia in patients with bone tumors. The study obtained the ethical approval from the studied hospital’s ethics committee (SLKYLX2023-171). All participants provided written informed consent and voluntarily took part in the study.

### 2.1 Study participants

All study participants were recruited using convenience sampling. The inclusion criteria were: (1) a diagnosis of primary or metastatic bone tumors; (2) a disease duration of at least 3 months; and (3) age 18 years or older. The exclusion criteria included: (1) presence of lower limb edema; (2) mental disorders; (3) severe hearing, vision, or speech impairments; (4) inability to stand; (5) presence of metallic implants; (6) limb amputations; (7) limbs wrapped in bandages or casts; and (8) use of medications that could affect body composition measurements.

### 2.2 AWGS 2019 sarcopenia diagnosis

According to the 2019 AWGS criteria (7), sarcopenia is diagnosed based on three key components: (1) Muscle Strength: Defined as reduced grip strength, with thresholds of < 28 kg for men and < 18 kg for women; (2) Muscle Mass: Assessed using bioelectrical impedance analysis (BIA), with an icular skeletal muscle mass index of < 7.0 kg/m^2^ for men and < 5.7 kg/m^2^ for women; (3) Physical Performance: Evaluated by gait speed, where a speed of < 1 m/s over a 6-m walk indicates impaired performance.

### 2.3 Data collection tools

#### 2.3.1 General information sheet

This information sheet was developed based on a comprehensive literature review to collect demographic and clinical information. This included details such as age, gender, education level, marital status, icular skeletal muscle mass index, body mass index, calf circumference, grip strength, and gait speed.

#### 2.3.2 SARC-F questionnaire

The SARC-F, developed by Malmstrom and Morley (14), assesses five domains: strength, assistance in walking, rising from a chair, climbing stairs, and fall history. Each item is scored from 0 to 2, with the total score ranging from 0 to 10. A score of ≥ 4 suggests a high risk of sarcopenia. The scale was translated into Chinese: its reliability (Cronbach’s alpha = 0.849), and the criterion-related validity (*r* = 0.878) (15).

#### 2.3.3 SARC-Calf questionnaire

The SARC-Calf integrates the SARC-F with calf circumference measurements developed by Barbosa-Silva et al. (16). For males with a calf circumference ≤ 34 cm or females with ≤ 33 cm, 10 points are added to the SARC-F score. The total possible score ranges from 0 to 20, with a score of ≥ 11 indicating a high risk of sarcopenia.

#### 2.3.4 SARC-F + EBM questionnaire

The SARC-F + EBM, developed by Kurita et al. (17), combines the SARC-F with an expanded body mass index (EBM) assessment, incorporating calf circumference measurements. A total score of ≥ 11 points denote a high risk of sarcopenia.

#### 2.3.5 MSRA-5 and MSRA-7 questionnaires

The Mini Sarcopenia Risk Assessment (MSRA) was developed by Rossi et al. (18), and later translated into Chinese by Yang et al. (19). The MSRA-5 consists of five items: age, history of hospital stays, physical activity level, daily meals, and weight loss, with a total score of ≤ 45 indicating sarcopenia risk. The MSRA-7 expands on this by including dairy and protein consumption, and a score of ≤ 30 suggests a sarcopenia risk.

### 2.4 Data collection

One nurse researcher (the first author) collected the data. Within 24 h of admission, eligible patients were informed about the study, and written consent was obtained. They were then asked to complete the five questionnaires, with assistance provided if needed. Calf circumference was measured three times while patients were seated with their dominant leg exposed, and the average of the three measurements was recorded. Before treatment, assessments of grip strength, walking speed, and icular skeletal muscle mass index were conducted. Grip strength was measured using an electronic grip strength meter (Xiangshan EH-101) with the dominant hand, or with the unaffected hand for patients with arm tumors, and the highest value from three attempts was recorded. Walking speed was measured twice over a 6-m distance, and the average speed was calculated. The icular skeletal muscle mass was assessed using an InBody 770 body composition analyzer.

### 2.5 Statistical analysis

Data were analyzed using SPSS version 25.0. Continuous variables were presented as means with standard deviations, while categorical variables were reported as frequencies and percentages. Receiver operating characteristic (ROC) curves were generated based on the 2019 sarcopenia diagnostic criteria, and the area under the ROC curve (AUC) along with 95% confidence intervals (CI) were calculated. For each screening tool, sensitivity, specificity, positive predictive value (PPV), negative predictive value (NPV), kappa value, and their respective 95% CIs were compared. The DeLong test was employed to evaluate differences between the AUCs of the screening tools. Youden’s index was calculated to identify optimal cutoff points. A *p* < 0.05 was considered statistically significant.

## 3 Results

### 3.1 Characteristics of study participants

This study included 300 bone tumor patients, aged 19–81 years, with a median age of 58 years (mean = 55.18, SD, 14.28 years). Based on the AWGS 2019 sarcopenia diagnostic criteria, the prevalence of sarcopenia among bone tumor patients was 26%. The difference of characteristics of patients with sarcopenia and without sarcopenia was presented in Table 1.

**TABLE 1:** General characteristics of Chinese bone tumor patients (*N* = 300).

Variable		Non-sarcopenic(*n* = 222)	Sarcopenic (*n* = 78)	U/t/χ^2^	*p*-value
Gender	Men	112	42	0.266	0.606
	Women	110	36		
Age (years)		56.47 ± 12.27	63.86 ± 10.60	3.53	0.001
GSa (kg)	Men	30.38 ± 6.51	23.37 ± 6.06	6.06	<0.001
	Women	19.94 ± 4.04	15.06 ± 5.15	5.19	<0.001
GSb (m/s)		1.08 ± 0.09	0.94 ± 0.14	8.71	<0.001
ASMI (kg/m^2^)	Men	8.00 ± 0.64	6.65 ± 0.48	15.45	<0.001
	Women	6.54 ± 0.58	5.41 ± 0.30	15.13	<0.001
CC (cm)	Men	33.45 ± 2.94	30.19 ± 2.67	6.54	<0.001
	Women	32.91 ± 2.52	28.90 ± 3.16	6.92	<0.001
BMI (kg/m^2^)		23.54 ± 3.13	19.80 ± 2.21	11.46	<0.001
Educational level	Primary school and below	96	49	9.25	0.026
	High school	63	13		
	College	37	8		
	University and above	24	8		
Marital status	Single	17	9	3.22	0.35
	Married	197	67		
	Divorce	5	0		
	Widower	3	2		

ASMI, icular Skeletal Muscle Mass Index; BMI, Body Mass Index; CC, Calf circumference; GSa, Grip strength; GSb, Gait speed.

### 3.2 Accuracy analysis of five sarcopenia screening tools

Using the AWGS 2019 diagnostic criteria as a reference, we assessed the sensitivity, specificity, positive predictive value, negative predictive value, and AUC for five sarcopenia screening tools. The results are summarized in Table 2.

**TABLE 2:** Accuracy analysis of five screening tools for sarcopenia based on AWGS 2019 (*N* = 300).

Screening tools	Sensitivity (%)	Specificity (%)	PPV (%)	NPV (%)	AUC (95%CI)
SARC-F	32.05	93.69	64.10	79.69	0.79 (0.73–0.85)
SARC-Calf	80.77	81.08	60.00	92.31	0.85 (0.81–0.90)
SARC-F + EBM	44.87	95.50	77.78	83.14	0.88 (0.84–0.92)
MSRA-7	74.36	65.32	42.03	87.88	0.77 (0.71–0.83)
MSRA-5	74.36	62.16	40.28	87.34	0.78 (0.72–0.84)

SARC-F, strength, assistance with walking: rise from a chair, climb stairs and falls; SARC-Calf, SARC-F combined with calf circumference; EBM, elderly and body mass index information; MSRA-5, mini sarcopenia risk assessment-5; MSRA-7, mini sarcopenia risk assessment-7.

### 3.3 Optimal cutoff values for the five sarcopenia screening tools

**FIGURE 1:**
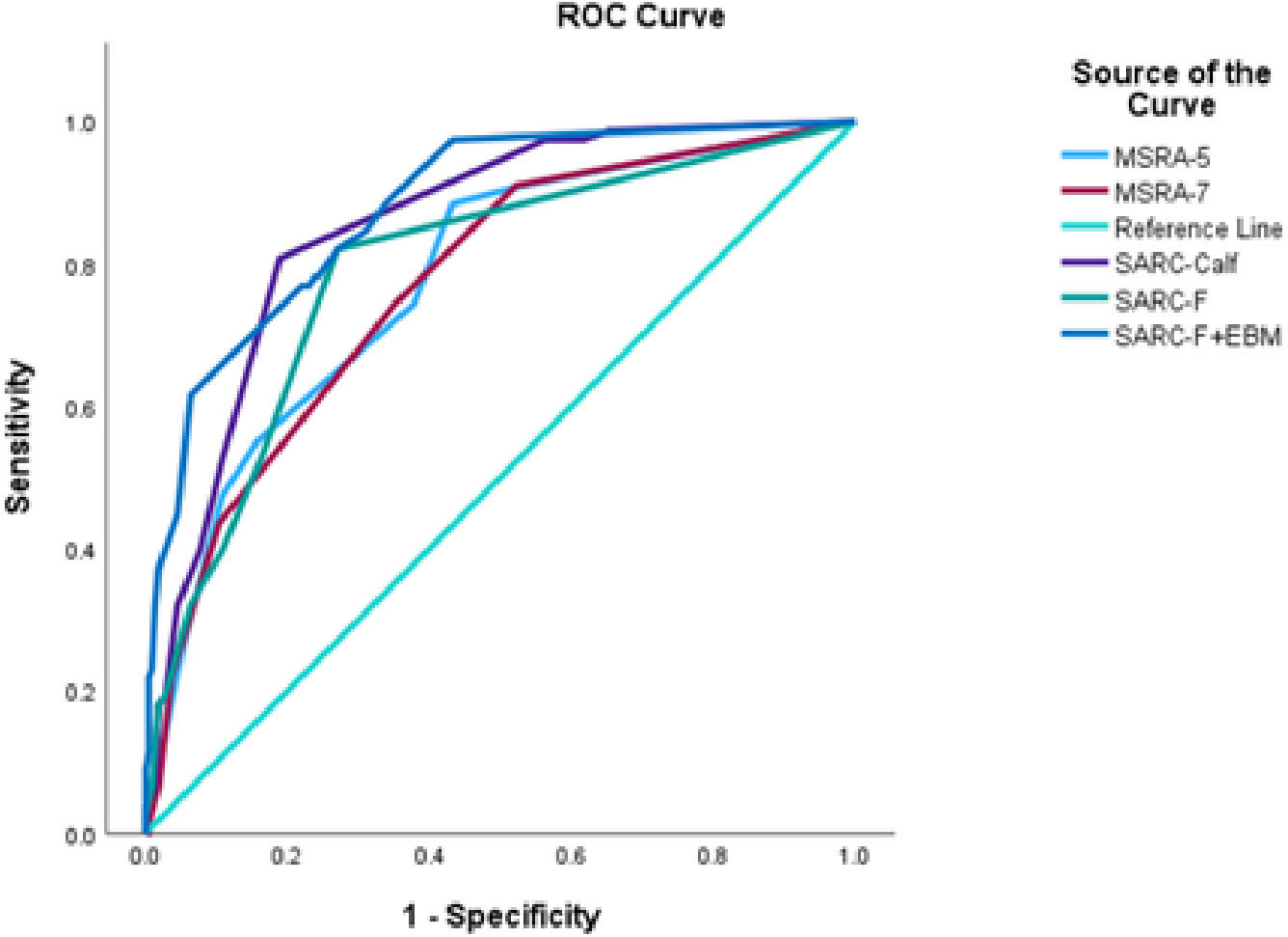
Comparisons of five screening tools for sarcopenia based on AWGS 2019. SARC-F, strength, assistance with walking, rise from a chair, climb stairs and falls; SARC-Calf, SARC-F combined with calf circumference; EBM, elderly and body mass index information; MSRA-5, mini sarcopenia risk assessment-5; MSRA-7, mini sarcopenia risk assessment-7.

Among the five screening tools, the SARC-F + EBM questionnaire showed the highest AUC (0.88) with an optimal cutoff point of 10.5. The ROC curve is shown in Figure 1. At this cutoff, the sensitivity and specificity were 61.5 and 93.7%, respectively (Supplementary Table 1).

### 3.4 AUC Comparison between the five screening tools

The AUC comparison revealed that the SARC-F + EBM questionnaire had a significantly higher AUC of 0.88 than the SARC-F, MSRA-7, and MSRA-5 questionnaires. Similarly, the SARC-Calf questionnaire had a higher AUC of 0.85 than the SARC-F, MSRA-7, and MSRA-5 questionnaires. There were no significant differences in AUC between the other tools (Table 3).

**TABLE 3:** The results of pairwise comparison of the area under the ROC curve of 5 screening tools (*N* = 300).

Screening tools	*Z*-value	*p*-value
SARC-F—SARC-Calf	−3.84	0.001
SARC-F—SARC-F + EBM	−3.13	0.002
SARC-F—MSRA-7	1.41	0.159
SARC-F—MSRA-5	0.43	0.651
SARC-Calf—SARC-F + EBM	−1.60	0.228
SARC-Calf—MSRA-7	3.49	< 0.001
SARC-Calf—MSRA-5	2.52	0.012
SARC-F + EBM—MSRA-7	4.51	< 0.001
SARC-F + EBM—MSRA-5	3.39	0.001
MSRA-7—MSRA-5	−2.10	0.035

ARC-F, strength, assistance with walking; rise from a chair, climb stairs and falls. SARC-Calf, SARC-F combined with calf circumference; EBM; elderly and body mass index information; MSRA-5, mini sarcopenia risk assessment-5; MSRA-7, mini sarcopenia risk assessment-7.

### 3.5 Comparison of sarcopenia screening results of the five tools with AWGS 2019 sarcopenia diagnostic criteria

Based on the screening results of the five tools, patients were classified into sarcopenia and non-sarcopenia groups. A comparison with the AWGS 2019 diagnostic criteria showed that the Kappa values for the SARC-F, SARC-Calf, SARC-F + EBM, MSRA-7, and MSRA-5 questionnaires were 0.307, 0.556, 0.468, 0.321, and 0.288, respectively (all *P* < 0.001), indicating moderate agreement (Table 4).

**TABLE 4:** Comparison of screening results of 5 sarcopenia screening tools with AWGS 2019 diagnostic criteria for sarcopenia (*N* = 300).

Screening tools		AWGS 2019 diagnostic criteria for sarcopenia (*n*)		Total (*n*)	Kappa value	*p*-value
		Yes	No			
SARC-F	( + )	25	14	39	0.31	< 0.001
	(−)	53	208	261		
	Total	78	222	300		
SARC-Calf	( + )	63	42	105	0.56	< 0.001
	(−)	15	180	195		
	Total	78	222	300		
SARC-F + EBM	( + )	35	10	45	0.47	< 0.001
	(−)	43	212	255		
	Total	78	222	300		
MSRA-7	( + )	58	77	138	0.32	< 0.001
	(−)	20	145	165		
	Total	78	222	300		
MSRA-5	( + )	58	84	144	0.29	< 0.001
	(−)	20	138	158		
	Total	78	222	300		

SARC-F, strength, assistance with walking, rise from a chair, climb stairs and falls; SARC-Calf, SARC-F combined with calf circumference; EBM, elderly and body mass index information; MSRA-5, mini sarcopenia risk assessment-5; MSRA-7, mini sarcopenia risk assessment-7.

### 3.6 Combined use of SARC-F and MSRA screening tools

**FIGURE 2:**
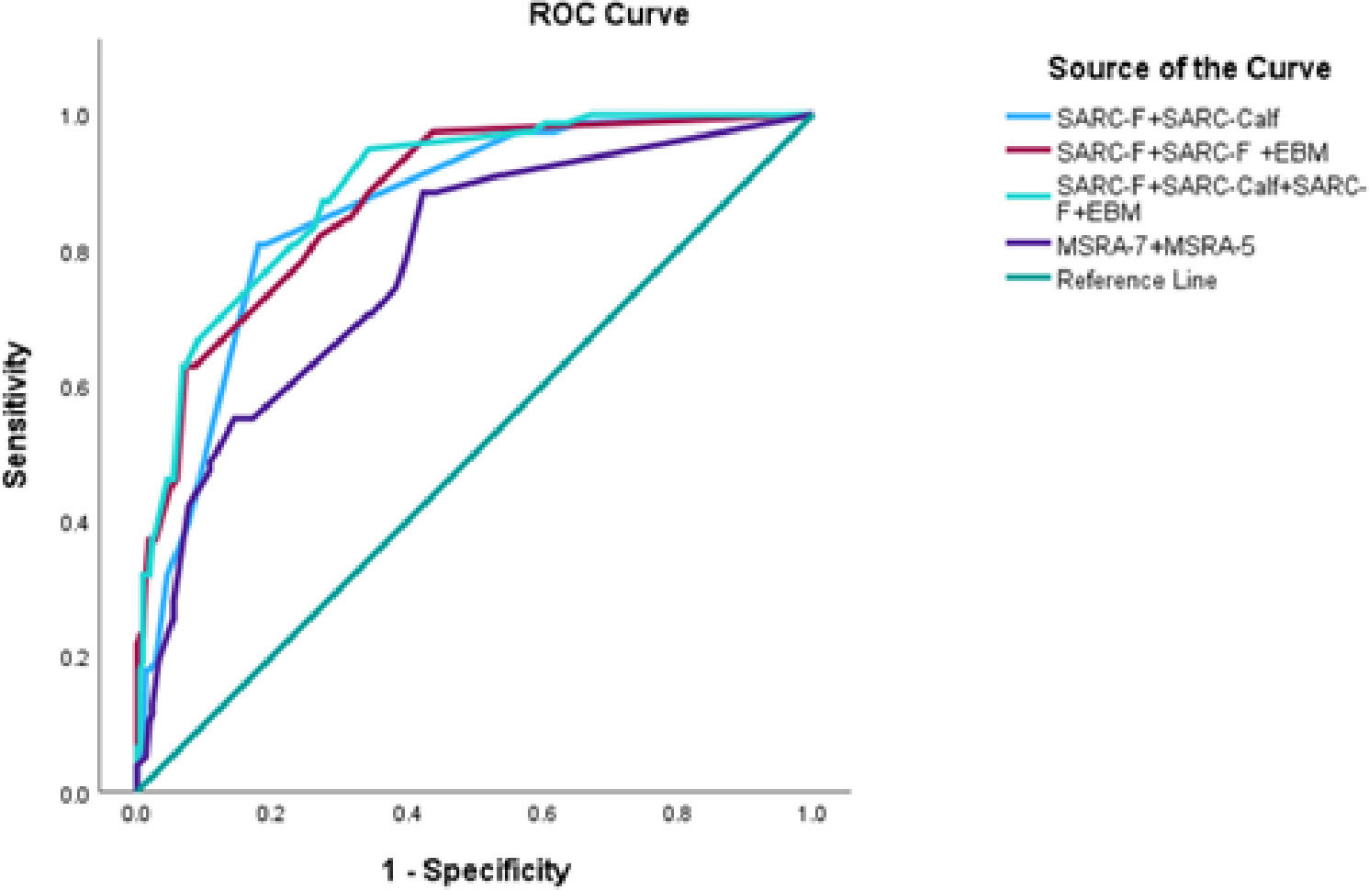
Comparisons of combined use of screening tools for sarcopenia based on AWGS 2019. SARC-F, strength, assistance with walking, rise from a chair, climb stairs and falls; SARC-Calf, SARC-F combined with calf circumference; EBM, elderly and body mass index information; MSRA-5, mini sarcopenia risk assessment-5; MSRA-7, mini sarcopenia risk assessment-7.

When the SARC-F and SARC-Calf tools were used together, the AUC under the ROC curve was 0.86. Combining SARC-F with SARC-F + EBM resulted in an AUC of 0.88. Using all three tools (SARC-F, SARC-Calf, and SARC-F + EBM) together further increased the AUC to 0.89. In contrast, when MSRA-7 and MSRA-5 were combined, the AUC was 0.78. The corresponding ROC curves are shown in Figure 2, and results are summarized in Table 5.

**TABLE 5:** Comparison results of combined use of SARC-F related scales and MSRA related scales (*N* = 300).

Screening tools	Youden index	Sensitivity (%)	Specificity (%)	AUC
SARC-F + SARC-Calf	0.63	80.8	82.0	0.86 (0.81–0.90)
SARC-F + SARC-F + EBM	0.56	62.8	92.8	0.88 (0.83–0.92)
SARC-F + SARC-Calf + SARC-F + EBM	0.61	94.9	94.1	0.89 (0.85–0.93)
MSRA-7 + MSRA-5	0.45	88.5	89.2	0.78 (0.72–0.84)

SARC-F, strength, assistance with walking, rise from a chair, climb stairs and falls; SARC-Calf, SARC-F combined with calf circumference; EBM, elderly and body mass index information; MSRA-5, mini sarcopenia risk assessment-5; MSRA-7, mini sarcopenia risk assessment-7.

## 4 Discussion

This study, using the AWGS 2019 sarcopenia screening criteria (20), found that the prevalence of sarcopenia in bone tumor patients was 26%, higher than previous similar studies with a prevalence of 22% (21–23). This may be due to bone tumors compressing spinal cord nerves, leading to paralysis and reduced physical activity (24). Additionally, bone tumor patients are less likely to experience early-stage digestive dysfunction compared to patients with gastrointestinal cancers, where insufficient nutritional intake increases the risk of sarcopenia (4, 25). Although the findings differ across studies, they highlight that sarcopenia in bone tumor patients is a significant concern (21, 22).

Most research on sarcopenia focuses on older adults, but bone tumor patients include younger individuals. The lack of awareness about sarcopenia, coupled with its complex screening process and the high costs of assessment, makes it challenging to screen these patients effectively (13). Therefore, healthcare professionals should emphasize early screening and prevention of sarcopenia in bone tumor patients to delay its onset and mitigate complications. Given the unique risks in bone tumor patients, Distinguishing sarcopenia from cachexia is essential, as they are distinct but can coexist, particularly in cancer settings (26, 27). Future research should address this overlap to refine screening and intervention strategies for all age groups affected by bone tumors.

In this study, the SARC-F questionnaire had high specificity but low sensitivity, consistent with previous research, indicating its limited ability to detect sarcopenia, leading to a high misdiagnosis rate. This is because SARC-F mainly reflects muscle strength and physical performance but does not assess muscle mass, a key diagnostic criterion for sarcopenia (28). The ROC analysis revealed an optimal cutoff value of 0.5, which aligns with Ma et al.’s findings (29), but differs from other studies (30, 31) due to regional and population differences. Adjusting the cutoff improved the sensitivity and balanced specificity, enhancing the screening performance of SARC-F for bone tumor patients. This suggests that clinicians need to determine optimal cutoff values tailored to specific populations to maximize the effectiveness of screening tools.

The SARC-Calf questionnaire, which incorporates calf circumference, showed higher sensitivity and specificity compared to SARC-F, offering better diagnostic value (Kappa value of 0.556). However, it still demonstrated low PPV and high NPV, consistent with previous research (32). Calf circumference is a reliable marker of skeletal muscle mass (33) and has been validated in diagnosing sarcopenia among older adults (7), patients with chronic liver diseases (34), and stroke survivors (35). However, the optimal cutoff value varies by study and population. For example, studies in Turkey and Taiwan found cutoffs of 33 cm for men and 32–33 cm for women (7, 36), while Japanese researchers recommend 34 cm for men and 33 cm for women (37). These variations may stem from differences in ethnicity, culture, and lifestyle (38). The AWGS2019-recommended cutoffs used in this study were appropriate for this population.

The SARC-F + EBM questionnaire, which adds age and BMI to the SARC-F tool, had higher sensitivity, specificity, and overall diagnostic performance than SARC-F alone, consistent with previous research (17). This is particularly meaningful for elderly patients with bone tumors, who are at greater risk of muscle loss due to reduced mobility and long-term bed rest. Moreover, studies have shown that low BMI (≤ 21 kg/m^2^) is associated with undernutrition, which increases the risk of sarcopenia (39, 40). Bone tumor patients often experience malnutrition due to chemotherapy-related gastrointestinal side effects and cachexia, further increasing sarcopenia risk. With an AUC of 0.88, the SARC-F + EBM tool demonstrated strong screening performance.

The MSRA-7 questionnaire showed high sensitivity but lower specificity and a lower PPV. Among the five screening tools, it had the smallest AUC (0.77). One possible explanation is that patients undergoing chemotherapy may have been hospitalized frequently during the past year, affecting their scores, even though their condition may have improved post-chemotherapy. Furthermore, the inclusion of daily dairy consumption as a factor may not align with typical Chinese dietary habits, impacting the tool’s performance. After removing dairy and protein consumption items, the MSRA-5 questionnaire performed better in terms of sensitivity and specificity, though its agreement with diagnostic outcomes was still suboptimal. Future research should aim to revise the MSRA tool for better applicability in bone tumor patients in China.

When SARC-F-related scales and MSRA-related scales were used in combination, the diagnostic performance improved. Specifically, the combined use of SARC-F, SARC-Calf, and SARC-F + EBM yielded the best diagnostic results, offering a comprehensive evaluation of muscle strength, physical performance, muscle mass, age, and nutrition. This combination provides a more practical and efficient method for early screening of sarcopenia in bone tumor patients.

Bone tumors and sarcopenia are interrelated. The disease itself, alongside treatments and psychological stressors, increases the prevalence of sarcopenia in bone tumor patients. Nurses play a crucial role in early detection, assessment, and intervention, making it essential to incorporate validated and practical sarcopenia screening tools into routine nursing assessments. By identifying patients at risk of sarcopenia early, nurses can implement tailored interventions that focus on maintaining or improving muscle strength, nutritional status, and overall physical function. This proactive approach can enhance postoperative recovery, reduce the severity of chemotherapy side effects, shorten hospital stays, and lower healthcare costs among bone tumor patients.

Additionally, the study highlights the importance of individualized patient education. Nurses can educate bone tumor patients and their families about the risks of sarcopenia, the importance of maintaining muscle mass through proper nutrition and physical activity, and how to recognize early signs of sarcopenia. These efforts can empower patients to take an active role in their care, potentially improving outcomes and quality of life in bone tumor patients. The study underscores the need for further research to validate and refine sarcopenia screening tools specifically for bone tumor patients, considering the unique characteristics of this population.

### 4.1 Study limitations

This study compared the effectiveness of five sarcopenia screening tools in bone tumor patients, identifying the best tools for early detection and providing a basis for prevention and further research. Although these tools were primarily developed for elderly populations, the inclusion of younger patients (≥ 18 years old) highlighted that sarcopenia can also affect non-elderly individuals. This emphasizes the need for healthcare professionals to pay greater attention to sarcopenia in bone tumor patients. However, the study has several limitations. First, the sample was small and drawn from a single hospital. Future studies should expand the sample size and include multicenter surveys of different types and stages of bone tumors to enhance the predictive power of these tools. Second, the study did not conduct longitudinal follow-up or evaluate the predictive value of the tools for adverse outcomes. Third, while BIA was used for muscle measurement, more accurate methods, such as CT, MRI, or dual-energy X-ray, could provide more precise assessments. Furthermore, the study focused on a specific cancer type and a particular ethnic group, which may limit the applicability of the findings to other cancer types or ethnicities. Finally, this study is the use of single-center, convenience-based sampling and the cross-sectional nature of the study, which may limit the generalizability of the findings.

### 4.2 Study implications

This study identified the SARC-F + EBM questionnaire and the combined use of SARC-F, SARC-Calf, and SARC-F + EBM as the top-performing tools for sarcopenia screening in bone tumor patients. The SARC-F + EBM demonstrated exceptional diagnostic performance, with high sensitivity, specificity, and an AUC of 0.88, making it highly effective for early detection. The combined approach further improved accuracy by comprehensively assessing muscle strength, physical performance, muscle mass, age, and nutritional status. For practical implementation, healthcare professionals should prioritize early screening using these validated tools in clinical settings, particularly for bone tumor patients at risk due to reduced mobility or malnutrition. Nurses should be trained to incorporate these tools into routine assessments to facilitate timely intervention. Additionally, individualized patient education on maintaining muscle mass through nutrition and physical activity is essential to improve outcomes. Further research is recommended to refine these tools for bone tumor patients and adapt them to diverse populations.

## 5 Conclusion

The study concludes that among these five screening tools for sarcopenia in bone tumor patients, the SARC-F + EBM and SARC-Calf methods exhibit higher accuracy than the SARC-F, MSRA-7, and MSRA-5 tools, for identifying sarcopenia in patients with bone tumors. These two tools are recommended as the most effective for clinical healthcare professionals in screening for sarcopenia. Furthermore, the combined use of SARC-F, SARC-Calf, and SARC-F + EBM yields even greater screening efficacy.

## Data Availability

The raw data supporting the conclusions of this article will be made available by the authors, without undue reservation.
