# Supplementary material for: Comparative assessment of sarcopenia screening tools for patients with bone tumors: insights for enhanced clinical application
Source: Front Nutr. 2025 Aug 4;12:1584706. doi: 10.3389/fnut.2025.1584706 (PMC12358746; doi:10.3389/fnut.2025.1584706)
Supplement: Supplementary file 1 [file Table_1.docx]

**Supplementary Table. Analysis of the optimal cut-off value of five screening tools for sarcopenia based on AWGS 2019** (N=300)

| Screening tools | Youden index | Best cutoff value | Sensitivity (%) | Specificity (%) |
| --- | --- | --- | --- | --- |
| SARC-F | 0.55 | 0.5 | 82.1 | 72.9 |
| SARC-Calf | 0.62 | 10.5 | 80.8 | 88.1 |
| SARC-F+EBM | 0.55 | 10.5 | 61.5 | 93.7 |
| MSRA-7 | 0.39 | 32.5 | 74.4 | 64.8 |
| MSRA-5 | 0.45 | 42.5 | 88.5 | 56.7 |

Abbreviations:

SARC-F: strength, assistance with walking, rise from a chair, climb stairs and falls. SARC-Calf: SARC-F combined with calf circumference.

EBM: elderly and body mass index information.

MSRA-5: mini sarcopenia risk assessment-5.

MSRA-7: mini sarcopenia risk assessment-7.
